# Use of a Guided Imagery Mobile App (See Me Serene) to Reduce COVID-19–Related Stress: Pilot Feasibility Study

**DOI:** 10.2196/32353

**Published:** 2021-10-04

**Authors:** Judith S Gordon, David Sbarra, Julie Armin, Thaddeus W W Pace, Chris Gniady, Yessenya Barraza

**Affiliations:** 1 College of Nursing University of Arizona Tucson, AZ United States; 2 Department of Psychology University of Arizona Tucson, AZ United States; 3 Department of Family and Community of Medicine College of Medicine University of Arizona Tucson, AZ United States; 4 Department of Computer Science University of Arizona Tucson, AZ United States

**Keywords:** COVID-19, stress, anxiety, isolation, intervention, guided imagery, mobile app

## Abstract

**Background:**

The SARS-CoV-2 pandemic has led to concerns about mental health resulting from regional and national lockdowns, social isolation, job loss, and concern about disease exposure.

**Objective:**

We describe results of the pilot feasibility study of the See Me Serene mHealth app. The app provides users with immersive, vivid, nature experiences to reduce stress and anxiety related to COVID-19 and other isolation. The goals of the study were to develop the See Me Serene app and test the feasibility and acceptability of study procedures, and explore the potential impact of the app on stress and anxiety.

**Methods:**

We developed and tested the See Me Serene app and our study procedures for feasibility, and gathered preliminary data with a goal of 100 participants. The research was conducted in 2 phases: (1) development and internal testing of the app; and (2) feasibility and pilot testing with participants recruited online through earned media (eg, news stories), presentations at a university campus, and social media (eg, online sharing of earned media and presentations). The feasibility study employed a mixed methods, within-subjects, pre-/posttest design. At baseline and 30-day follow-up, we assessed stress-related variables via validated self-report measures and saliva samples for determination of cortisol concentrations.

**Results:**

We met or surpassed all our feasibility benchmarks for recruitment (101 participants recruited), retention (91% [90/99] of 30-day assessment completed), and data collection (99 participants completed all baseline data; 85% [84/99] of salivary cortisol samples returned). Participants adhered to the intervention. On average, participants listened to 48.2 audio files over 30 days or approximately 1.6 audio files per day. Participants were satisfied with the app, with 87% (78/90) rating the app as helpful in dealing with stress and anxiety. The app showed the potential to reduce stress, anxiety, loneliness, and worry. We did not find significant differences (*P*=.41) in cortisol levels over time. Our findings suggest that future research is warranted to test the efficacy of the See Me Serene app with a representative, diverse sample.

**Conclusions:**

There is a need for evidence-based and easily disseminable stress-reduction interventions. See Me Serene is a feasible intervention and has the potential to reduce stress related to COVID-19 and other forms of social isolation. More research on See Me Serene is warranted.

## Introduction

The SARS-CoV-2 pandemic is a major social upheaval associated with considerable lifestyle changes and economic burdens around the globe—including lockdowns, social isolation, and job loss. The pandemic has hastened concerns about mental illness and emotional distress worldwide, especially among frontline and essential workers who are directly impacted by disease exposures and the psychological sequelae of these potential exposures [1]. A recent meta-analytic work suggested that the prevalence of major depressive disorder has increased substantially since the inception of the pandemic [2]. New evidence suggests that rates of loneliness, the subjective experience of social isolation, have increased dramatically during the COVID-19 pandemic [2-4]. The psychological consequences of long-term home confinement and quarantine are extensive, including anxiety, stress, and insomnia, among other aversive states [5]. It is well known that these psychological states have biological correlates [6] and stress-related psychological changes (eg, disruption of diurnal cortisol rhythm) are associated with risk for a range of poor health outcomes, including cancer, heart disease, and metabolic disease [7]. The need to address the behavioral health consequences of COVID-19 is urgent.

Cortisol is a hormone commonly measured as a biomarker of stress [8]. Diurnal cortisol rhythm is the fluctuation of circulating cortisol over the course of the day, which can be assessed by measuring cortisol concentrations in saliva [9]. Saliva concentrations of cortisol are highest in the morning just before waking, and lowest in the evening before bedtime. Greater cortisol concentrations are available in the morning when it is needed to initiate key physiological processes (eg, metabolism, central nervous system arousal). Diurnal cortisol rhythm is distinct from other patterns of cortisol secretion (eg, phasic responses to acute stress challenge, or a meal). Relative disruptions in diurnal cortisol rhythm (ie, less change over the course of the day) have been associated with chronic conditions (eg, asthma, obesity, and major depression) [7], while a more dynamic change in saliva concentrations of cortisol from morning to evening has been associated with improved physical well-being [10].

Guided imagery can reduce anxiety, chronic pain, and change lifestyle behaviors [11-23]. Guided imagery is unique from mindfulness meditation in that it is an immersive experience in which participants use all their senses and emotions to imagine a scene or situation. In our previous studies, we delivered guided imagery interventions using digital health technologies, including an mHealth app (*See Me Smoke-Free;* NCI, grant number R21CA174639) for promoting smoking cessation, healthy eating, and physical activity, and via telephone (*Be Smoke Free*; NCCIH, grant number R34AT008947) [24,25]. Guided imagery for stress reduction may appeal to both those who embrace other forms of meditation for relaxation and those who use guided imagery for improving athletic performance [26,27].

Stay-at-home orders, lockdowns, and other forms of social isolation may remove people from nature and the outdoors [28]. Several recent reviews indicate that exposure to nature has a positive effect on stress, depression, and anxiety [29-31]. In a systematic review of 12 randomized controlled trials and cross-sectional studies, Shuda and colleagues [31] found that the exposure to nature reduced both self-reported stress (as measured by the Perceived Stress Scale [PSS] and other validated instruments) and physiologic stress (as measured by salivary cortisol, blood pressure, functional magnetic resonance imaging, skin conductance, heart rate variability, and other standardized measures). In a literature review of 113 articles, Berto [29] summarized that exposure to natural scenes mediates the negative effects of stress and the cognitive deficits associated with stress, and improves mood. Corazon and colleagues [30] conducted a systematic review of 36 studies conducted between 2012 and 2018 on the effects of outdoor, nature-based exposure on stress. They found that interventions focused on outdoor exposures showed strong evidence for positive outcomes on perceived stress, while the results for physiologic measures were more varied [30]. Overall, the studies contained in these reviews indicate that there is evidence to support the use of outdoor imagery to reduce perceived and physiologic stress [29-31]. The definition of outdoor, nature-based experiences is broad. Therefore, they could include more traditional nature scenes (eg, mountains, beach) as well as more “adventure-oriented” scenes (eg, playing golf, riding a bicycle) which may be relaxing to specific individuals.

Although technologies exist to provide immersive experiences (eg, virtual reality), they require specialized equipment and can be cost-prohibitive. Combining guided imagery with smartphone technology is a low-cost, scalable, and disseminable method for allowing people to engage in evocative, immersive experiences in nature—from sitting in a forest to cycling on a bike path. The vast majority of Americans across age, race/ethnicity, and socioeconomic status own smartphones; and of those, most use mobile apps [32,33]. Several studies have shown that people experiencing stress are more likely to use meditation apps (eg, Calm) [34-37]. Using a simple mHealth app to deliver guided imagery audio files has the potential for simulating outdoor experiences for people experiencing lockdowns and social isolation, as well as for individuals who are homebound due to other reasons.

Therefore, we developed and tested the feasibility and potential impact of the *See Me Serene* mHealth app. See Me Serene provides users with immersive, vivid, nature experiences designed to reduce stress and anxiety related to social isolation. Herein, we describe the results of this pilot feasibility study.

## Methods

### Study Overview

We conducted the pilot study between May 1, 2020, and December 31, 2020. The study was approved by the University of Arizona Institutional Review Board (Protocol #2005625231). The primary goals of the study were to develop the See Me Serene app and test our study procedures for feasibility. Our secondary goal was to gather preliminary data regarding the potential impact of the app on reducing perceived and physiologic stress; the study was not designed nor powered to test efficacy. The research was conducted in 2 phases: (1) development and internal testing of the app; and (2) feasibility and pilot testing with participants recruited online through earned media (eg, new stories), presentations on a university campus, and social media (eg, online sharing of earned media and presentations). The feasibility study employed a mixed methods, within-subjects, pre-/posttest design. Feasibility outcomes included metrics for participant recruitment (goal of 100 within 2 months), participant retention (at least 75% at 1 month), and data collection (at least 90% at baseline and 75% at the 1-month follow-up). Exploratory outcomes included changes in self-reported measures of stress, anxiety, loneliness, and worry, and diurnal cortisol rhythm (collected via salivary cortisol assays) from baseline to 1-month follow-up. We also collected consumer satisfaction at the 1-month assessment.

### Intervention Development and Description

#### Program Development

In Phase 1, we developed and conducted internal testing on the See Me Serene app. The app was developed based on feedback regarding guided imagery for relaxation obtained in a previous study [25]. Initial guided imagery scripts were reviewed by the investigators and project staff and vetted by an advisory board that consisted of faculty, staff, and students at the University of Arizona. During this process, the advisory board suggested the addition of outdoor activities to relaxing nature scenes to enhance the appeal of the app to a more diverse population of users and differentiate See Me Serene from other relaxation apps in the marketplace. The app was programmed by undergraduate students in the Department of Computer Science with oversight by the investigators (CG and JG). We developed Android and iOS versions of the app using the Thunkable platform, which allows simple design and app development. This process streamlined development and accelerated deployment by automatically generating native apps for both Android and iOS platforms. We used Google’s Firebase which provides database and authentication services targeted for mobile apps. The app audio and image files were stored in the Firebase database along with user preferences. This allowed for easier updates of guided imagery files without requiring app updates. Beta testing of the See Me Serene app was conducted by the project team and members of the Advisory Board. Once complete, the app was deployed to the App Store and the Google Play Store. See Me Serene was updated twice during the study to fix bugs and make improvements to usability.

#### See Me Serene Description

The See Me Serene app allows users to choose from a variety of guided imagery audio files and associated photos of immersive, evocative nature scenes. Users can select audio files from guided imagery categories such as “outdoor adventures” (eg, motorcycling along a country road, hiking in the desert, fishing) and “serene scenes” (eg, in the mountains, at the lake, at the beach). Each file contains detailed, vivid descriptions of the scenarios, including sights, sounds, smells, tastes, tactile sensations, and emotions. Each audio file starts with a brief relaxing breathing exercise and instructions to “release any tension in your body and mind.” The audio files in the app, which were tested during this study, included soft background music and were approximately 5 minutes in length. Users receive notifications to listen to the files once each day. See Me Serene tracks the user’s mood each time the user logs in with 4 questions assessing how often/much the user has felt stressed, anxious, lonely, or worried “today” (0=not at all to 4=very often/extremely). The app tracks and displays the user’s profile information and the number of guided imagery audio files listened to. See Me Serene provides a list of clickable links to free or low-cost, evidence-based, mental health resources, some of which are available 24/7. The app also contains a list of frequently asked questions and answers addressing common technical or content-related questions, and an About Us page describing the study team.

### Feasibility Pilot Study

In Phase 2, we conducted a within-subjects, pre-/posttest, pilot feasibility study to recruit a convenience sample of 100 participants recruited nationally through earned media (eg, news stories), presentations on a university campus, and social media (eg, sharing of news stories and presentations online). Recruitment took place between August 3, 2020, and October 5, 2020. Potential participants were eligible for the study if they were over aged 18; had a valid phone number and email address; owned a smartphone with internet access; and spoke English. Potential participants were excluded if they had ever been told by a health care provider that they had an adrenal gland disorder such as Cushing disease or Addison disease, or if they regularly used any corticosteroid medications such as hydrocortisone, prednisone, or fludrocortisone acetate. Recruitment of the convenience sample took place via earned media (ie, stories carried by local, regional, and national news outlets on television, radio, and the internet), presentations at a university campus, and social media (ie, reposting of earned media stories and presentations). Recruitment materials described guided imagery and the See Me Serene app in general terms and invited potential participants to visit the project website for more information or download the app from the App Store or the Google Play Store. After signing into the app, users were given the option to participate in the study or just use the app.

Participants completed a baseline assessment online or by phone. We also collected salivary cortisol (a biomarker of stress) samples pre- and posttest in the home setting. Saliva sample kits were sent to participants by overnight mail. Participants were instructed to collect 2 samples per day (1 in the morning immediately after waking, and 1 at night right before bed) for 2 consecutive days, and record the time of day when they collected their samples on an instructions form that was included in the saliva sample kits. Participants were instructed to not brush their teeth, eat, exercise, smoke, take a cold shower, or consume alcohol or caffeine until after they had collected a saliva sample. We also instructed participants to not collect a sample until at least 60 minutes after eating dinner in the evening. After samples were collected, participants stored them in their home freezer until the posttest, at which time they repeated the collection process and returned all 8 samples and instructions form with collection times to the research team by prepaid, overnight mail. Study staff provided reminders to participants to collect samples at both baseline and follow-up.

Participants were instructed to use the app at least once each day for 4 weeks. Follow-up assessments (online or telephone-delivered self-report surveys and salivary cortisol assays) were conducted at 30 days after access to the app. Use of the app was collected automatically within the Firebase database (in which specific app use data were stored), and through Google Analytics and Apple Developer Tools (eg, app downloads and current users). Upon completion of all study activities, participants received a US $50 electronic gift card.

### Assessments

#### Data Collected

At baseline, we collected demographics including age, gender, race, ethnicity, education, marital status, number of people and pets in the household, mental health diagnoses, and amount of moderate to vigorous physical activity per week. We also collected data on COVID-19–related variables including stay-at-home orders, essential personnel status, length of restrictions, leave the house every day status, amount of time spent outdoors, and amount of time spent doing fun outdoor activities.

At baseline and 30-day follow-up, we assessed stress-related variables via validated self-report measures and saliva for determination of cortisol concentrations.

#### Perceived Stress Scale (PSS)

Self-reported stress was measured via the PSS [38]. The PSS is a 10-item scale that assesses the perception of psychological stress and the degree to which people feel events in their life are unpredictable and uncontrollable, as well as the extent to which the events tax their coping resources over the past month. Each item is scored (or reverse scored from 0 to 4), and the total score is summed across all items. Population norms for the PSS are mean (SD) 12.1 (5.9) for males and 13.7 (6.6) for females. The PSS has excellent psychometric properties and is widely used.

#### Overall Anxiety Severity and Impairment Scale (OASIS)

The Overall Anxiety Severity and Impairment Scale (OASIS) is a 5-item scale for assessing anxiety symptoms associated with multiple anxiety disorders [39]. Each item is measured on a 5-point Likert scale (0=none/no anxiety to 4=constant/extreme/all the time anxiety) with respondents reflecting on their symptoms in the past week. Participants rate how often they have felt anxious in the past week, the intensity of their anxiety, their degree of avoidance of situations and activities, and the degree of interference in daily activities and social relationships. The items are summed for a total score. In the validation research, a score of 8 or greater correctly classified 87% of the sample as having an anxiety disorder or not [40].

#### Impact of Events Scale—Revised (IES-R)

The Impact of Events Scale—Revised (IES-R) [41] assesses subjective emotional distress, typically over the prior 7 days, following a traumatic or stressful event. Our focus was on the COVID-19 pandemic. Example items include statements such as, “Any reminder brought back feelings about it,” and “I feel irritable and angry.” The scale has 22 questions using a 5-point Likert scale rating system ranging from 0 (Not at all) to 4 (Extremely). The total IES-R has high internal consistency, and the measure is widely used in the literature. On the IES-R, a cutoff score of 1.5 (or a total score of 33) provides the highest diagnostic power with a sensitivity of 0.91 against the Posttraumatic Stress Disorder (PTSD) checklist [41].

#### University of California Los Angeles Loneliness Scale

The 3-item University of California Los Angeles (UCLA) Loneliness Scale [42] includes the following items, “How often do you feel you lack companionship?” “How often do you feel left out?,” and “How often do you feel isolated from others?.” The items are scored on a 3-point Likert scale from “hardly ever” to “often.” This scale has excellent psychometric properties and is widely used in the literature.

#### The Brief Penn State Worry Questionnaire (PSWQ)

The ultra-brief Penn State Worry Questionnaire (PSWQ) [43] was used to measure worry and consists of 3 validated items on a 5-point scale, with individual items scored between 0 and 5 and a composite score of 0-15 (0=no worry and 15=high worry). The items focus on the situations that make people work, the extent of the worry, and the controllability of worry. In a treatment-seeking sample, the mean of the brief PSWQ was 10.42 (SD 3.44).

#### Cortisol

Finally, we assessed diurnal rhythm changes over time in cortisol, a biomarker of stress, via saliva concentrations of cortisol determined with enzyme immunoassay kits (obtained from Salimetrics), according to manufacturer instructions. Samples were assayed in duplicate per sample, and the lower limit of sensitivity of the assay was 0.006 μg/dL. Intra- and interassay coefficients of variation of the cortisol enzyme immunoassay were 9.6% and 4.7%, respectively.

#### Guided Imagery and Other Meditation

We also measured the use of guided imagery (number of minutes per day in the last week); use of breathing, meditation, or progressive relaxation (number of minutes per day in the last week); and 2 items adapted from the Borkovec and Nau Credibility/Expectancy Questionnaire [44] regarding perceived confidence in guided imagery to reduce stress, and perceived logic for the use of guided imagery to reduce stress (rated on a 5-point Likert scale, where 1=very unconfident/very illogical to 5=very confident/very logical) [24,25].

#### Physical Activity

Exercise (past week) was measured using the 3-item Godin Leisure-Time Exercise Questionnaire (LTEQ) [45,46]. The LTEQ is sensitive enough to differentiate mild, moderate, and strenuous exercise, can be scored in total number of minutes of activity or as a metabolic equivalents, and differentiates between household, occupational, and leisure-time physical activity and seasonal variations [47].

#### User Satisfaction

At the 30-day follow-up, we also assessed usability and participant satisfaction with the app. We assessed 6 items of usability, including: (1) “How organized was the app?”; (2) “How much did you like the format/design of the app?”; (3) “How easy was the app to use?”; (4) “How well did the app address issues you have with anxiety/stress?”; (5) “How much new information did this app provide?”; and (6) “How helpful did you find the features of this app?”

We asked 3 satisfaction items: (1) “Overall, how would you rate this app in helping you deal with stress/anxiety?”; (2) “How likely would you be to recommend this app to other people?”; and (3) “How likely are you to continue using this app?” We also elicited open-ended feedback about the app with the question, “What suggestions do you have for improving the app?”

#### App Use

We automatically collected use of the app, including number of guided imagery audio files listened to and number of times answering the daily questions. We also tracked the number of downloads and current users from Google Analytics and Apple Developer Tools.

#### Feasibility

Finally, we assessed feasibility outcomes by achievement of our feasibility benchmarks, including meeting the recruitment/accrual goal of 100 participants within 2 months; collection of at least 90% of baseline self-report data; collection of at least 75% of follow-up self-report surveys and cortisol samples; and at least 75% participant retention at 30 days.

### Analyses

Study data were collected and managed using REDCap electronic data capture tools hosted at The University of Arizona [48,49]. REDCap (Research Electronic Data Capture) is a secure, web-based software platform designed to support data capture for research studies, providing (1) an intuitive interface for validated data capture; (2) audit trails for tracking data manipulation and export procedures; (3) automated export procedures for seamless data downloads to common statistical packages; and (4) procedures for data integration and interoperability with external sources. Descriptive statistics were compiled using the REDCap reports and stats function. Our feasibility analyses focused on change in the main psychological outcome variables over time using paired-samples *t* tests. Open-ended responses to the question, “What suggestions do you have to improve the app?” were summarized using a matrix analysis approach, which is an efficient method of descriptively organizing and interpreting qualitative findings for program integration [50]. For diurnal cortisol rhythm, we computed the slope of the change in cortisol from morning to afternoon to evening for each collection day, taking into consideration the time of sample collection [51]. In cases where sample collection time was missing, average sample collection time was used [51]. We then averaged cortisol slopes across both collection days to develop a metric of diurnal cortisol rhythm.

## Results

### Participant Characteristics

We recruited 101 participants from August 3, 2020, to October 5, 2020. Following enrollment, 2 participants withdrew from the study (1 due to technical issues and 1 due to a family emergency), leaving 99 participants with complete data at baseline. As displayed in Table 1, the majority of participants were non-Hispanic White (83/99, 84%) females (85/99, 86%) who had at least some college education (93/99, 94%), and were married or living with a partner (66/99, 67%). The average age of participants was 51.4 (SD 14.7) years. COVID-19 restrictions affected 68% (67/99) of participants, and of those under stay-at-home orders, 92% (61/66) reported that they had been staying at home for more than 3 months. Participants reported diagnoses of generalized anxiety disorder (24/99, 24%), major depression (18/99, 18%), and PTSD (12/99, 12%). Participants had an average score of 3.03 (SD 0.54) on the PSS; 8.05 (SD 4.03) on the OASIS; 2.29 (SD 0.58) on the IES-R; 2.73 (SD 0.77) on the UCLA Loneliness Scale; and 8.94 (SD 3.15) on the PSWQ. Most participants reported using less than 10 minutes of guided imagery (90/99, 91%) or other relaxation techniques (73/99, 74%) per day in the previous week. Still, they expressed confidence in the use of guided imagery for reducing stress (86/99, 87%) and found it logical to use guided imagery for stress reduction (93/99, 94%).

**Table 1:** Baseline participant characteristics^a^.

Characteristics	Values^b^
Age (years), mean (SD)	51.4 (14.7)
Female sex	85 (86)
Non-Hispanic White	83 (84)
Hispanic ethnicity	17 (17)
At least some college	93 (94)
Married or living with a partner	66 (67)
Generalized anxiety disorder	24 (24)
Major depression	18 (18)
Posttraumatic stress disorder	12 (12)
At least two people in household	79 (80)
At least one pet in household	68 (69)
Currently under stay-at-home order or restricting where you go	67 (68)
Staying at home for more than 3 months (N=66)	61 (92)
Not exempt from stay-at-home orders	73 (74)
Leave house each day (sometimes to often)	61 (62)
Go outdoors (sometimes to often)	86 (87)
Do fun outdoor activities (sometimes to often)	53 (54)
Get <3 hours of moderate/vigorous physical activity per week	65 (66)
Perceived Stress Scale score, mean (SD)	19.16 (4.58)
Overall Anxiety Severity and Impairment Scale score, mean (SD)	8.05 (4.03)
Impact of Events Scale—Revised score, mean (SD)	2.29 (0.58)
University of California Los Angeles Loneliness Scale score, mean (SD)	2.73 (0.77)
Brief Penn State Worry Questionnaire score, mean (SD)	8.94 (3.15)
Morning cortisol (µg/dL), mean (SD)	0.33 (0.36)
Evening cortisol (µg/dL), mean (SD)	0.05 (0.03)
Cortisol slope, mean (SD)	–0.43 (0.47)
<10 minutes per day in the past week practiced guided imagery	90 (91)
<10 minutes per day in the past week practiced other relaxation techniques	73 (74)
Confidence in guided imagery to reduce stress (somewhat to very)	86 (87)
Logical to use guided imagery to reduce stress (somewhat to very)	93 (94)

^a^N=99 unless otherwise noted.

^b^Values shown are n (%) unless otherwise noted.

### Feasibility

As displayed in Table 2, we met or surpassed all our feasibility benchmarks. We were able to recruit 100 participants within 2 months, collected 98% (99/101) of baseline survey data, 91% (90/99) of 30-day surveys, and 85% (84/99) of cortisol samples were returned. Five samples contained some missing data and were excluded from analyses for a total of 80 (79/99) usable samples. Using a conservative definition of retention (complete survey and cortisol data at 30 days), we surpassed our goal with an 85% (84/99) retention rate.

**Table 2:** Feasibility outcomes.

Outcome	Value, n/N (%)	Feasibility criteria	Feasible?
Participant recruitment, n	101	Enroll 100 participants in 2 months	Yes
Baseline surveys	99/101 (98)	Collect at least 90% of survey data	Yes
30-Day surveys	90/99 (91)	Collect at least 75% of survey data	Yes
Cortisol samples returned	84/99 (85)	Collect at least 75% of cortisol data	Yes
Participant retention (conservatively defined as those with complete data at 30 days)	84/99 (85)	At least 75% participant retention at 30 days	Yes

### Adherence

Adherence was measured by the number of times participants listened to guided imagery audio files during the 4-week intervention period. On average, participants listened to 48.2 audio files (range 0-280) over 30 days, or approximately 1.6 audio files per day. This finding establishes the feasibility of listening to at least one audio file per day as instructed. We also found a significant increase in time spent each day using guided imagery (t_89_=–9.94, *P*<.001) and other forms of meditation (t_89_=–7.34, *P*<.001).

### Usability and Satisfaction

Overall, the 90 participants retained at the 30-day follow-up found the app usable and were satisfied with it. See Table 3 for individual ratings of usability and satisfaction. Suggestions for improving the app included increasing audio file length, providing more variety in the guided imagery scenarios (eg, more nature-focused guided imagery), customizing the narrator’s voice and background music or sounds, allowing users to save their favorite audio files to a library within the app, and providing users the ability to track their use of the app and easily return to where they were.

**Table 3:** Participant ratings (somewhat to very) of usability and satisfaction with the app (N=90).

Question	Values, n (%)
How organized did you find this app?	88 (98)
How much did you like the format/design of the app?	84 (93)
How easy was the app to use?	88 (98)
How well did the app address issues you have with anxiety/stress?	81 (90)
How much new information did the app provide?	66 (73)
How helpful did you find the features of this app?	80 (89)
Overall, how would you rate this mobile app in helping you deal with stress/anxiety?	78 (87)
How likely would you be to recommend this app to others?	71 (79)
How likely are you to continue using this app?	66 (73)

### Potential Impact on Outcome Measures

As shown in Table 4, we found preliminary evidence that the See Me Serene app may have the potential to reduce anxiety. Although this study was not designed to test efficacy, we found significant reductions in all outcome variables with small to medium effect sizes. Participants reported reductions in self-reported stress (t_89_=3.38, *P*=.001), symptoms of PTSD (t_89_=4.02, *P*<.001), anxiety (t_89_=3.80, *P*<.001), loneliness (t_89_=3.74, *P*<.001), and worry (t_89_=2.77, *P*=.007) from pre- to posttest. We did not find significant differences (*P*=.41) in cortisol levels over time.

**Table 4:** Changes in outcome variables pre-/post test.

Measure	Preintervention, mean (SD)	Postintervention, mean (SD)	*t*	*df*	*P*	Cohen *d*
Perceived Stress Scale score	19.16 (4.58)	17.5 (5.11)	3.38	89	.001	0.37
Overall Anxiety Severity and Impairment Scale score	8.05 (4.02)	6.63 (3.44)	3.80	89	<.001	0.40
Impact of Events Scale—Revised score	2.29 (0.58)	2.01 (0.51)	4.02	89	<.001	0.42
University of California Los Angeles Loneliness Scale score	2.73 (0.77)	2.51 (0.85)	3.74	89	<.001	0.40
Brief Penn State Worry Questionnaire score	8.95 (3.15)	8.82 (2.79)	2.77	89	.007	0.29
Cortisol (morning)	0.33 (0.36)	0.48 (1.35)	–0.99	79	.32	–0.11
Cortisol (evening) average	0.06 (0.08)	0.14 (0.62)	–1.25	80	.21	–0.14
Average cortisol slope	–0.44 (0.47)	–0.54 (1.22)	0.81	79	.41	0.09

## Discussion

### Principal Results

#### Feasibility

The results of this study suggest that the See Me Serene app is feasible to deliver and test. Our results appear to be similar to other studies that have found mHealth apps feasible to deliver and easy to disseminate [52-55]. We met or surpassed all of our feasibility benchmarks, including the recruitment and retention of participants and the collection of both self-report data and biochemical samples. Our ability to easily recruit participants solely through earned media indicates the great need for novel, easily disseminable, stress-reduction interventions. Our high retention and data collection rates suggest that participants are eager to help find solutions for managing stress. Finally, we found that it was feasible to conduct the study entirely remotely, and to collect biological samples even during a pandemic when the logistics of mailing and receiving shipments were challenging.

Participants were adherent to the study protocol and listened to an average of 2 files each day, surpassing the minimum requirement of listening to 1 audio file per day. Our results compare favorably with other studies that have shown modest adherence or engagement with mHealth stress management apps [52,55,56]. Based on participant feedback, our adherence results may have been due in part to the length of the guided imagery audio files because each file was approximately 5 minutes long. The length of the guided imagery was based on those used in our previous studies [24,25]. However, participants expressed a desire for longer audio files, and they may have listened to multiple files or the same file multiple times each day to achieve this result. Participants significantly increased the number of minutes per day in the last week that they practiced guided imagery as well as other forms of relaxation, such as meditation, deep breathing, or progressive muscle relaxation. The guided imagery did include a brief breathing exercise at the beginning of each audio file but did not focus solely on breathing, nor did the audio files teach meditation or progressive muscle relaxation. Therefore, it appears that the use of this app may have encouraged users to seek out and engage in other forms of relaxation techniques as well.

#### Usability and Satisfaction

Overall, participants found See Me Serene easy to use and useful. Our results were similar to previous studies of mHealth apps aimed at reducing stress, anxiety, and depression among a variety of different populations [55-59]. There were few reported technical issues, which were resolved with updates to the app. Although 87% (78/90) of participants found the app helpful in reducing stress and would recommend the app to others, only 73% (66/90) reported that they would continue to use the app. This may have been due to several factors: (1) stay-at-home orders were lifted during the study, allowing for less isolation; (2) easing of restrictions may have reduced the stress faced by the participants, thus they had less need for the app; (3) the app was effective at reducing participants’ stress, thus reducing their need to use the app; (4) participants may have wanted changes to the app to enhance their experience, as suggested by the qualitative data.

The open-ended question on the follow-up survey asking for suggestions provided the team with input regarding app revisions and offered insight into how the app was being used by participants, which can help us tailor revisions to their needs. For instance, several participants noted that they used the audio files before bed to relax for sleep. These individuals generally wanted longer audio files to include music that would continue after the guided imagery stopped. In general, the comments supported longer guided imagery files that allowed listeners to practice breathing and develop the mental imagery described in the narration. This recommendation may result from participants’ lack of familiarity with guided imagery (eg, only 9% [9/99] reported using guided imagery more than 10 minutes a day at baseline), and the revision could make the app content more accessible to users who are not familiar with guided imagery. Participants also wanted more variety of guided imagery, and the ability to personalize the app to their needs. Providing more and different types of guided imagery, and features such as the ability to choose the narrator’s voice, save favorites, and track mood and audios listened to over time may improve engagement with the app and encourage continued use.

#### Potential Impact

Although this study was not designed to test efficacy, we found significant reductions in all outcome variables with small to medium effect sizes. The evidence regarding effectiveness of mHealth apps is limited and mixed [53,54,56-64]. While several randomized controlled trials have shown these apps to be effective at reducing stress, anxiety, depression, or PTSD symptoms [53,54,57,59], some have not reported positive results [60,61,64]. In addition, many studies report only significant pre–post results [56,58,62,63], thus limiting generalizability of the findings. Finally, several reviews have found that the majority of currently available mHealth apps are not evidence based, and lack a strong theoretical basis and research to support efficacy [65-68].

Although our study was not designed to test efficacy, we found significant reductions in all outcome variables with small to medium effect sizes. Participants in our study reported reductions in self-reported stress, symptoms associated with PTSD and anxiety, loneliness, and worry. However, this was an open within-subjects design which did not have a control group, and we recruited a convenience sample. Therefore, participants knew the purpose of the app and that it used guided imagery. For example, participants may have been more inclined to use guided imagery versus other types of stress reduction methods. At baseline, almost all participants reported confidence in and logic of guided imagery to manage stress.

We did not find significant differences in cortisol levels over time. This could be because of the relatively heterogeneous sample with respect to age and baseline psychological well-being characteristics, as well as the possibility that life stressors associated with the pandemic (ie, social isolation, limited time outdoors, myriad sources of psychological distress) may have prevented the intervention from exerting an effect on diurnal cortisol rhythm that could be detected across the whole sample, especially in the short term. Controlling or stratifying for psychological and physical well-being and relevant sources of stress, including later assessment time points and increasing the sample size, would all likely help reveal intervention efficacy at the level of diurnal cortisol rhythm [9,10]. However, it is possible that a “light touch” intervention such as ours may not produce immediate physiologic changes in cortisol. This variability in physiologic outcomes is consistent with the results of the systematic review by Corazon and colleagues [30], suggesting the need for more research. Although our findings are promising, more research is needed to determine whether See Me Serene is effective at reducing stress-related symptoms and objective measures of stress-related biology.

### Limitations

This study had several limitations. First, we used a within-subjects design without a control group, which limited our ability to test the feasibility of an active control condition as well as to determine efficacy as a result of specific (as opposed to nonspecific) intervention components. The research team has already developed a control app that will serve as an active attention comparator, and a pilot study is planned. In addition, we were not powered to detect differences in mood or other outcomes based on usage of different types of audio files (eg, “adventure scenes” versus “relaxing scenes”). In the future, we plan to conduct a fully powered efficacy trial which will allow for subanalyses. Second, we recruited a convenience sample of participants, which limits the generalizability of the findings. Participants were primarily college educated, White, and women. The lack of diversity may have resulted from reliance on earned media (eg, news stories) and presentations on a university campus due to budget constraints. Future studies will employ methods aimed at recruiting a more diverse sample, including more males and racial/ethnic groups. We will actively recruit low-income and racial/ethnic minority participants to demonstrate feasibility and usability of the See Me Serene app with these groups. We have used these methods successfully in previous trials to yield a more representative sample. Third, we followed participants for only 1 month. Subsequent studies will follow participants for longer periods to assess the duration of the intervention effect. We have successfully followed participants for 6 months in our previous trials [24,25]. Fourth, this study was conducted during the COVID-19 pandemic, which caused some delays and logistic challenges with the collection and return of the saliva samples to determine cortisol concentrations. However, overall, these challenges (eg, managing overnight shipping, providing replacement saliva collection supplies) were overcome, with the result being a large percentage of saliva samples collected and returned in a manner acceptable for laboratory analysis. Finally, we could not collect data from the 9 participants who dropped out of the study regarding why they did not complete the study or what they thought about the app. However, a 9% (9/99) dropout rate is very low compared with that of many mHealth studies which often have higher dropout rates than in-person studies.

### Conclusions

The study results suggest that delivering and testing the See Me Serene mHealth app is feasible and that the app has the potential to reduce stress related to COVID-19 and other forms of social isolation. We learned valuable lessons during the pilot that can be applied to future studies. There is a great need for evidence-based and easily disseminable stress-reduction interventions, as the majority of available stress management apps are not evidence based. More research is warranted to test the efficacy of See Me Serene.

## Abbreviations

IES-R: Impact of Events Scale—Revised
LTEQ: Leisure-Time Exercise Questionnaire
OASIS: Overall Anxiety Severity and Impairment Scale
PSS: Perceived Stress Scale
PSWQ: Penn State Worry Questionnaire
UCLA: University of California Los Angeles
